# Stakeholder perspectives of care for people living with dementia moving from hospital to care facilities in the community: a systematic review

**DOI:** 10.1186/s12877-019-1220-1

**Published:** 2019-07-31

**Authors:** Angela Richardson, Alison Blenkinsopp, Murna Downs, Kathryn Lord

**Affiliations:** University of Bradford, Bradford, UK

**Keywords:** Dementia, Hospital, Care home, Transition, Family carers, Healthcare professionals, Discharge

## Abstract

**Background:**

People living with dementia in care homes are regularly admitted to hospital. The transition between hospitals and care homes is an area of documented poor care leading to adverse outcomes including costly re-hospitalisation. This review aims to understand the experiences and outcomes of care for people living with dementia who undergo this transition from the perspectives of key stakeholders; people living with dementia, their families and health care professionals.

**Methods:**

A systematic search was conducted on the CINAHL, ASSIA, EMBASE, MEDLINE, PsychINFO, and Scopus databases without any date restrictions. We hand searched reference lists of included papers. Papers were included if they focused on people living with dementia moving from hospital to a short or long term care setting in the community including sub-acute, rehabilitation, skilled nursing facilities or care homes. Titles, abstracts and full texts were screened. Two authors independently evaluated study quality using a checklist. Themes were identified and discussed to reach consensus.

**Results:**

In total, nine papers reporting eight studies met the inclusion criteria for the systematic review. A total of 257 stakeholders participated; 37 people living with dementia, 95 family members, and 125 health and social care professionals. Studies took place in Australia, Canada, United Kingdom (UK), and the United States of America (US). Four themes were identified as factors influencing the experience and outcomes of the transition from the perspectives of stakeholders; preparing for transition; quality of communication; the quality of care; family engagement and roles.

**Conclusion:**

This systematic review presents a compelling case for the need for robust evidence to guide best practice in this important area of multi-disciplinary clinical practice. The evidence suggests this transition is challenging for all stakeholders and that people with dementia have specific needs which need attention during this period.

**Trial registration:**

PROSPERO Registration Number: CRD42017082041.

## Background

People living with dementia are frequent users of hospital services [1], with bed occupancy rates as high as 25% at any one time [2]. The most common reasons for admission are injuries sustained from accidents and falls, urinary tract and respiratory infections and exacerbations of other chronic health problems [3]. For over a decade there has been considerable attention paid to hospital discharge processes, with particular emphasis on seeking to avoid unnecessary admissions and reduce delays in discharge, in order to alleviate pressure in the health system [4]. Yet people living with dementia continue to experience delayed hospital discharges and when discharged, feel inadequately prepared; therefore it is not surprising hospital readmissions are common [2].

Internationally, the transition between hospitals and care homes has been highlighted as a concern; with several studies noting care at this transition requires improvement [5–8]. In the UK, over two-thirds of care home residents are reported to be living with dementia [9]. Although information is limited about the use of hospitals by care home residents, Quality Watch, a UK major research programme, published a report following their analysis of hospital admission rates. Their findings imply that care home residents experienced 40–50% more emergency admissions than the rest of the population over the age of 75 years [10].

It is widely reported that transitions in care for older people lead to error, breaches of patient safety, rehospitalisation and mortality [11–13]. As a result, older people require additional resources in order to transfer safely from hospital in order to avoid these post-discharge adverse events [13, 14]. The components of care that are particularly found to be lacking during transfers to care homes for older people are patient and family engagement in planning for the transfer [5, 6]; timely prepared transfers [7]; follow up care [8]; and communication about health and medication between settings [6]. These can all lead to frequent hospital readmissions [15]. These studies were not particularly focused on the experiences of people living with dementia.

Internationally, different terminology is used to refer to the care provided when a person transfers out of hospital back to the community. Improving this care has received most attention in the US and Australia. The term ‘transitional care’ appears to have emerged in the US in the 1980’s [16]. It refers to a broad range of actions including proactive, collaborative planning, service identification, and follow-up activities delivered to ensure continuity of healthcare to improve patient outcomes when people move between levels or locations of care, most notably from the hospital to the community [17, 18]. The Australian government announced in 2004 the development of the ‘Transition care program’. This is a time limited initiative which enables older people with lower level support and care needs, to receive care in residential or community settings, to gain independence and confidence either to return home, or make decisions about moving into a more appropriate supported accommodation [19]. The concept of ‘transitional care’ is relatively new in the UK, where there appears to be a much narrower focus on discharge processes and planning. There is now recognition that discharge planning processes are time-limited by the length of hospital stay, from admission to the day of discharge [20] and are only one element of transitional care.

The bulk of transitional care research taking place in North America, Australia and Europe has been conducted with older people being discharged to their own homes, with more recent work including a focus on people living with dementia [21–23]. Where the transition between hospital and care home has been studied [5–8], people living with dementia are often not included. Despite the policy emphasis on person-centred care, we know relatively little about the experiences and outcomes of people directly affected - people living with dementia and their family carers, nor their health and social care professionals. A UK report has indicated that coordinated transitional care practice is variable for people living with dementia, who move between care settings [24]. There is now a compelling argument to review the literature, in order to understand more about the experiences and outcomes of this transition for people living with dementia.

The aim of this review is to identify and synthesise evidence from the published literature on the experiences and outcomes of care from hospital to a care home (or similar care facility) from the perspectives of people living with dementia, their families and health and social care professionals.

## Methods

This systematic review follows the guidance set out in the Centre for Reviews and Dissemination guidance for systematic reviews [25]. We registered the review with PROSPERO (International Prospective Register of Systematic Reviews) (PROSPERO 2017) [CRD42017082041].

### Search strategy

A CINAHL search strategy was developed without any date restrictions which was adjusted to run on five other electronic databases: ASSIA, EMBASE, MEDLINE, PsychINFO, and Scopus (See Table 1 for CINAHL strategy). The terms ‘Dementia’ or ‘Alzheimer’s disease’ or ‘cognitive impairment’ was used in combination with ‘transitional care’, or ‘discharge planning’, or ‘transfer, discharge’, and with ‘hospital’ or ‘care home’, or ‘residential care’ or ‘nursing home’ or ‘skilled nursing facility’. The search was run in April 2018. Additional papers were identified by hand searching the reference lists of included papers. No date restrictions were applied as we were interested in including relevant older studies. The oldest study retrieved for full text review was 1996 although this was excluded as it did not meet the inclusion criteria.

**Table 1:** (CINAHL search strategy)

	Search terms /combination	Results
1	(MH “Dementia+”)	(56,809)
2	(MH “Alzheimer’s Disease”)	(25,305)
3	“cognitive impairment”	(14,859)
4	1 OR 2 OR 3	(66,823)
5	(MH “Transfer, Discharge”)	(4,696)
6	(MH “Transitional Care”)	(482)
7	(MH “Discharge Planning”)	(4,403)
8	5 OR 6 OR 7	(9,359)
9	(MH “Hospitals+”)	(95,412)
10	(MH “Residential Care +”)	(6,320)
11	“care home”	(2,099)
12	(MH “Skilled Nursing Facilities”)	(2993)
13	(MH “Nursing Homes+”)	(23,732)
14	9 OR 10 OR 11 OR 12 OR 13	(124,718)
15	4 AND 8 AND 14	(76)

### Inclusion and exclusion criteria

This review focuses on both ‘experiences’ and ‘outcomes’ of transitional care. It was at times difficult to determine differences between ‘experiences’ and ‘outcomes.’ For this review, the team defined the term ‘experience’ as capturing the emotional and psychological responses to being involved in the transitional care process, for example feeling ‘unprepared’ or ‘frustrated’. ‘Outcomes’ related more to; effects, consequences and impact during or following the transition, for example ‘fully engaged in discharge planning’ or ‘communication failures’. Studies were included if they reported:

- The experiences and/or outcomes of care for people living with dementia or cognitive impairment (‘dementia’ and ‘cognitive impairment’ as defined by the authors of the individual studies), moving from hospital to a short or long term care setting (e.g. sub-acute, rehabilitation, skilled nursing facilities (SNF), care homes, including those returning to their care home after a hospital admission).
- Outcomes and experiences of the people living with dementia and/or their families were reported separately from those without cognitive impairment.
- Actions such as discharge planning from hospital to a care facility and any intervention or service people received during and following transition or the period waiting for long-term care placement.

Papers were excluded if:

- The majority of patients/residents referred to in the study were older people without cognitive impairment (we were led by the authors of the individual studies descriptions’ of ‘older’ and ‘cognitive impairment’).
- The majority of patients/residents were being discharged back to their own home (own home refers to a domestic setting, those returning back to their care home for example were not excluded)
- The study did not report on experiences or outcomes of care
- They were not written in English
- They did not report empirical findings that were published in peer review journals.

### Data extraction and quality appraisal

All references retrieved were exported to Endnote reference management software version X7 and duplicates removed. Titles and abstracts of studies were read and screened by one reviewer. Two reviewers then independently read all retained papers. The decision to include or exclude papers was agreed by consensus by both reviewers. A third reviewer was available if consensus could not be reached; recourse to a third reviewer was not required. Data were extracted from the included papers using a pre-determined set of criteria, which was developed by the review team after testing with a small number of papers. The final information extracted was, location, study setting, study type, study aim, study participants, main findings.

Each paper was assessed for quality independently by two reviewers, using checklists that had been developed and used by other authors [26] [27]. Only the checklists for qualitative and intervention papers were required for the current review. A point is awarded if the paper meets each criterion on the checklist, a maximum of six for qualitative and five for the intervention studies, high scores signifying higher quality (Table 2). Criteria were weighted to define higher quality studies as previously described in Lord et al. [27]. Qualitative papers were characterised as higher quality if they used a clearly defined recruitment method, had clearly stated inclusion and exclusion criteria, standardized data collection and involved two or more independent raters in data analysis (criteria 2,3,5). Intervention studies were characterised as higher quality if they appropriately allocated participants to intervention and control groups, ensured all participants who entered the trial were accounted for and collected data and followed up all participants in the same way (criteria1, 3, 4). Quality assessment was agreed by consensus; a third reviewer was available if consensus could not be reached, but was not required.

**Table 2:** Quality Assessment Tool. [27]

Quality assessment tool for Qualitative studies	
(1) Were the aims of the research clearly stated?	
(2) Was a clearly defined method of recruitment used and explicit inclusion/ exclusion criteria described?	
3) Was the process of data collection explained clearly? Was data collection standardised?	
4) Did the researchers attain saturation of data?	
(5) Was the process of data analysis sufficiently rigorous, i.e. ≥2 raters, some method of resolving discrepancies?	
6) Have the findings been validated by participants?	
Quality assessment tool for Intervention studies	
(1) Were participants appropriately allocated to intervention and control groups? (was randomisation independent?)	
(2) Were patients and clinicians as far as possible ‘masked’ for treatment allocation?	
(3) Were all patients who entered the trial accounted for and an intention to treat analysis used?	
(4) Were all participants followed up and data collected in the same way?	
(5) Was a power calculation carried out, based on one or more outcomes of interest?	

## Results

**Fig. 1:**
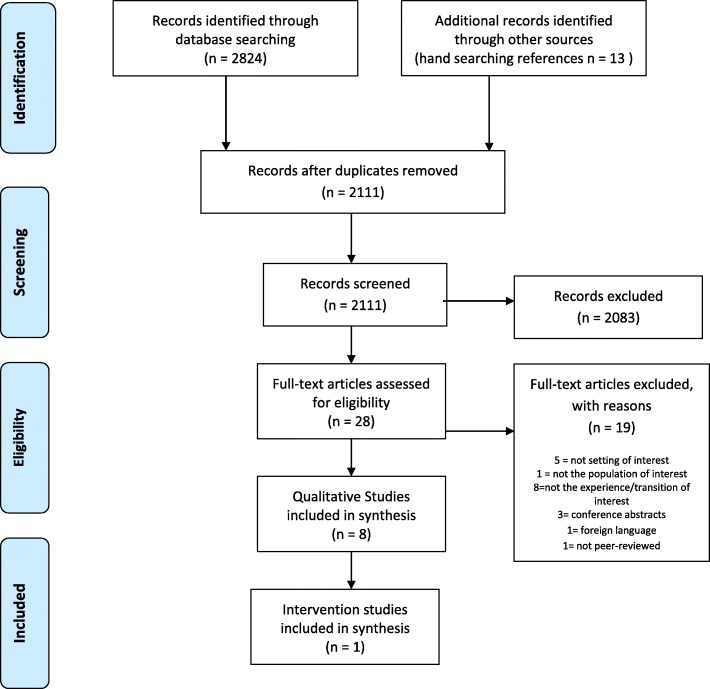
PRISMA Diagram

After removal of duplicate papers the number of retrieved titles and abstracts screened was 2111, and of those, 28 potentially met the inclusion criteria and were subject to full text scrutiny. Nine papers met the inclusion criteria (PRISMA Fig. 1). The majority of studies meeting the inclusion criteria used qualitative methodology. This may be due to the nature of the review question; focusing on perspectives. Eight of the papers reported qualitative studies and the ninth was a pre/post intervention evaluation. The nine studies are summarised in Table 3 (Study settings and service descriptions) and Table 4 (Study methods and quality appraisal).

**Table 3:** Description of settings/ services

Study and country	Study setting(s) / care service	Description or definitions of settings and or services	Transition points where perspectives are elicited
Bauer et al. (2011) [28] Fitzgerald et al. (2011) [29] Australia	Rehab facility (n = 8) Residential care (n = 8)	Rehabilitation facility, short-term restorative care before discharge back home or to residential care. Residential care – Long term care facilities providing high and low level care.	Family carers interviewed 2 months after discharge about their experiences.
Bloomer et al. (2016) [30] Australia	Geriatric evaluation and management facility	Provides rehabilitation to optimise function and determine future care needs. Majority of patients are transferred from acute care, a third of patients move to residential care.	Family carers of people with dementia were interviewed after admission into the Geriatric evaluation and management facility. Experiences were elicited about transitioning through the system from acute hospital.
Digby et al. (2012) [31] Australia	Geriatric rehabilitation facility (sub-acute facility)	A facility providing in-patient evaluation, and management of older patients with complex needs, most transferred from acute care setting.	People living with dementia interviewed between 1 and 5 days after transferring from hospital to the facility.
Emmett et al. (2014) [32] UK	Three general elderly care wards in two hospitals	Acute hospital care providing medical care for short-medium term acute episodes of care.	Patient and family carer interviews were conducted at point of discharge and 3 months post discharge. Health and social care perspectives elicited about discharge planning and decision-making.
Gilmore-Bykovsky et al. (2017) [33] USA	11 Skilled nursing facilities (SNF)	SNF’s provide high level of medical and nursing care. Services are provided for a limited time but can be more longer-term.	Nurses were interviewed about care when people had transitioned from hospital into the skilled nursing facility.
Kable et al. (2015) [34] Australia	Acute tertiary facility GP Practice Residential aged care setting	Acute hospital care Community care Long-term care facility.	Both hospital based and community based health care professionals’ perspectives of transitional care were elicited about care at the transition points of leaving hospital into the community.
Kuluski et al. (2017) Canada [35]	Hospital setting, (alternate level of care (ALC))	Patients who are fit for discharge but are waiting for long term care placement or community support.	Family carers perspectives were elicited whilst the patient was receiving the alternative level of care.
Renehan et al. (2013) [36] Australia	Transitional Care programme which was called ‘Transition Care Cognitive Assessment and Management Pilot’ (TC-CAMP)	Dedicated (short term) beds within a residential aged care facility, used specifically for people living with dementia who were medically fit to be discharged from hospital and would be transferring to long term care.	Health and social care professionals from all of the transition points; hospital, TC-CAMP and discharge destination care home. Family carers perspectives were gathered post discharge from the TC-CAMP.

**Table 4:** Table of studies, methods and quality appraisal

Study	Methods	Participants	Aims	Main findings	Quality appraisal					
					1	2	3	4	5	6
Bauer et al. 2011 [28] Fitzgerald et al. 2011 [29]	Semi-structured interviews	25 carers	Understand family carers experience of discharge planning, support, and what improvements could be made.	Breakdown in communication: lack of coordination, Hospital staff having poor capability for caring for people with dementia. Inadequate preparation, undervaluing family carer as a resource.	✓	✓	✓	✓	✓	✓
Bloomer et al. 2016 [30]	Semi-structured interviews / conversation approach	20 carers	Explore the experience of carers through hospitalisation and rehab with a view to transitioning to residential care.	Families found the process difficult. Decisions about moving into care was challenging, carers would like to be better informed, concerns about the care provided whilst in hospital.	✓	✓	✓	x	✓	x
Digby et al. 2012 [31]	Semi –structured interviews	8 people living with mild to moderate dementia, transferred in the preceding 5 days	Understand the experience of people living with dementia (plwd) who are settling in after transfer from acute hospital to sub-acute facility.	People felt disorientated. Participants felt patronised by staff and unsettled by the loss of control in the environment. Family support was a great consolation.	✓	✓	✓	x	x	x
Emmett et al. 2014 [32]	Ethnographic approach using observation, interviews and focus groups.	35 health and social care prof 29 patient interviews and cases 28 nominated relative	Explore the role of relatives during the discharge planning process and when decisions are made to discharge plwd from hospital either back home or to long-term care.	Roles relatives play; advocates, information gatherers, and care takers which included assisting. Lack of information inadequate preparation. Conflicts of interest between relatives and patients.	✓	✓	✓	✓	✓	✓
Gilmore-Bykovsky et al. 2017 [33]	Focus groups and semi structured interviews	40 licensed nurses from SNF’s	To examine SNF nurses’ perspectives regarding experiences and needs of plwd during hospital-to-SNF transitions.	Inadequate preparation of person, being excluded form care decisions. Unprepared receiving environment. Role of timing of transition. Inadequate information about social and health needs and behaviour related symptoms. Staff feeling ill-equipped to provide safe care. Misalignment between hospital pressures and transitional care needs of patient.	✓	✓	✓	✓	✓	✓
Kable et al. 2015 [34]	Focus groups	33 Health care professionals (HCPs) of which 21 hospital staff 12 community staff	Explore HCP perspectives on the discharge process and transitional care arrangements for plwd and their families.	Acute staff experienced difficulty caring for people with dementia. Patients were over sedated on return. System pressures to discharge. Inadequate preparation time for work capacity issues. Inadequate communication between health professionals working in different settings.	✓	✓	✓	x	✓	x
Kuluski et al. 2017 [35]	Semi structured interviews	15 family members across 12 interviews	Understand the hospital experience of carers of patients who require an Alternate Level of Care, (waiting for long-term placement).	Inconsistent quality of care, non-medical needs and characteristics ignored. Families addressing the gaps in the system. Confusing process.	✓	✓	✓	✓	✓	x
Renehan et al. 2013 [36]	Interviews, focus groups, file audits,	11 cases of which 8 had completed records, 7 family members took part in the qualitative evaluation 17 staff from the hospital, facility and destination facility	To evaluate the transitional care cognitive assessment management pilot. Identify barriers and enablers to implementation.	Significant reduction in agitated behaviours once moved to the transitional facility. Adequate communication provision and valued the clinical nurse consultant. Discharge destination facilities reported information timely and thorough.	Intervention					
					x	x	x	✓	x	

Studies were published between 2011 and 2017. Six of the papers were from Australia, [28–31, 34, 36] with two reporting findings from the same study [28, 29], one was from the US [33], one from the UK [32] and one from Canada [35].

Just two of the studies elicited perspectives of people living with dementia [31, 32]. Both authors interviewed people living with dementia; *N* = 8 and *N* = 29. Emmett et al. [32] additionally carried out ethnographic observations over 111 days. Renehan et al. [36] analysed the care records of people living with dementia to determine whether the transition programme was helpful to those residents with behavioural and psychological symptoms. Using interviews and or focus groups, five studies included the perspective of family members [28–30, 32, 35, 36] and four studies sought the views of health and social care professionals [32–34, 36]. The combined number of participants from each category was 37 people living with dementia, 95 family members and 125 healthcare professionals. A range of health and social care professionals were represented these included: medical staff working in hospitals and the community [32, 34]; allied health care professionals [32, 34, 36]; social workers, health care assistants and a professional advocate [32]. The largest professional group were nurses including those working in hospitals [32, 34, 36], community care facilities [33, 36] and in GP practices [34]. Staff working in residential care homes were included in two studies [34, 36]. Renehan et al’s [36] study also included a range of staff from the transition care programme which included personal assistants, diversional therapists, team leaders and managers.

Study settings where transitional care took place were varied but the majority were short-term stay settings. The description and definitions of settings or services and the transition points where the authors elicited perspectives can be found in Table 3. The majority of studies focused on the care provided at transition, including discharge planning, how decisions are made, and the processes involved in the transfer.

### Methodological quality

Seven of the qualitative studies were rated as higher quality, with scores ranging from four to six (Table 4). The one lower quality study score [31] reflects that insufficient information was given on the process of analysis and whether at least two raters had been involved. The pre/post intervention evaluation [36] was also rated as lower quality, meeting only one out of the five criteria, the low score relates to not having a comparison group and experiencing administrative issues resulting in incomplete records and measures only available for eight out of the 11 records.

### Narrative thematic analysis

The findings extracted from the papers were read multiple times. A constant comparative method of continually moving between the findings of each study was utilised. One reviewer coded the findings. These were grouped into related categories. A second reviewer checked these for accuracy. Four recurring themes pertaining to outcomes and experiences were identified from the included papers: preparing for transition; quality of communication; quality of care; carer engagement and roles of the family. Some themes were overlapping, for example preparation for transitions was closely linked to the quality of communication given when preparing for transfer. Quality of communication also linked to carer engagement. A summary of the experiences and outcomes from the different perspectives can be found in Table 5.

**Table 5:** Summary of experiences and outcomes from different perspectives

Stakeholders perspectives	Theme: Preparing for transition		Theme: Quality of communication		Theme: Quality of care		Theme: Family engagement and roles of family	
	Experiences	Outcomes	Experiences	Outcomes	Experiences	Outcomes	Experiences	Outcomes
People living with dementia.	Unable to remember preparation.	Disorientated by move.	Feeling unsettled and powerless. Feeling angry	Excluded from care decisions and decision making.	Feeling patronised and unsettled.	Lack of personal empowerment. Lack of understanding from staff.		Family support provides comfort.
Family carers	Insufficient preparation	Undermined ability to give informed opinion re planning. Lack of communication.	Feeling communication could be better. Appreciation of access to named professional.	Breakdown of communication between family and hospital. Care decisions made on insufficient information. HCPs fail to communicate adequately with person with dementia. Some reported adequate communication attending regular meetings. Difficulties getting hold of clinicians.		Assumptions made by HCPs about psychosocial needs of the people living with dementia. Concerns about standards of care. Some reported reduction in agitation, improved socialisation and health outcomes.	Feeling unappreciated and frustrated when excluded. Tension and family conflict about care decisions. Stressful experience leading up to discharge.	Families filling gaps in care system helping with hands on care and advocacy. Family support provides comfort to the person with dementia.
Health and Social care professionals (HCPs)	Person with dementia feeling stressed. HCPs feeling pressured. Unsettling for person with dementia.	Not preparing the person properly, unable to understand event. Quick transfers, insufficient time to prepare documentation. No time to organise environment and order equipment. Transfers late in day.	Stressful experience for person with dementia and family. Poor start to care home experience for person with dementia and family.	Care decisions made on insufficient information. HCPs fail to communicate adequately with person with dementia. People with dementia excluded from care decisions. Difficulties with individual care planning and providing care continuity. Affect the ability of the person to settle in new environment. Judgment of care facility as being inefficient. Some reported timely and comprehensive information.	Feeling ill-prepared and ill-equipped about how to care for people living with dementia.	People living with dementia returning to facility over sedated Not having original health care needs met. Under reporting of behavioural symptoms. Insufficient workforce to provide care.	Conflicts of interests between family members and person living with dementia.	Smoother transition when working with families. Working with family members could be difficult.

#### Preparing for transition

Preparing for the transition was discussed in five studies; by family carers [28, 29, 32], health and social care staff [33, 34] and briefly mentioned in one study reporting the experience of people living with dementia [31] . Perspectives were varied. The degree of feeling prepared was closely related to the quality of communication exchanges between stakeholders. For family members, being insufficiently prepared for being involved in discharge meetings undermined their ability to give an informed opinion about discharge decisions [32]. There were examples of carers feeling that the lack of communication relating to discharge arrangements left them feeling totally underprepared for their role, post transfer [28, 29]. One study reported the impact of not preparing the person properly contributed to a stressful experience for the person living with dementia: by allowing them to think they were being discharged home when in fact they were transferring to a care facility and by not allowing enough time to understand the decisions made [33]. People living with dementia in Digby et al’s study [31] were unable to recall the preparation involved, but many admitted to feeling disorientated by the move.

Hospital pressures to react to the increase in admissions meant that health and social care professionals felt additional pressure to discharge because the bed was required [33, 34]. This resulted in clinicians not being able to formulate detailed discharge documentation and transfers to care facilities being arranged quickly, without sufficient consultation. Insufficient time for the facility to prepare adequately, either in organising the environment, ordering specialist equipment, comfort items or medication were reported. There were also examples of transfers after hours, late at night which was neither conducive for the person with dementia or the receiving care facility. All of this militated against a successful transitional experience.

#### Quality of communication

Communication issues were reported by all stakeholders in all but one of the studies [35]. The quality of the communication between stakeholders was mostly described as inadequate. Breakdowns of communication between family members and the hospital were reported. Family carers felt that they could be better informed about the discharge planning, follow up care after discharge, and the options to assist with decision making during discharge planning [28].

Care decisions were often made on the basis of insufficient information of health status, care needs, dementia related behavioural symptoms, and the social history of the patient, which subsequently affected the quality of the transition for the person with dementia and or their family. This was reported both by families [28, 29] and by staff [33]. This proved difficult in areas such as individualised care planning, responding appropriately to the person with dementia and providing continuity. This was stressful for the person with dementia and their family, and could affect their ability to settle in the changed environment. Healthcare professionals also felt a poorly executed transition from hospital due to lack of information reflected badly on the care home who could be viewed as inefficient and disorganised, resulting in a poor start to the care home experience for both people living with dementia and their family [33].

Health and social care professionals fail to communicate adequately with the person with dementia about discharge planning and outcomes [31–33]. Examples were reported of people with dementia being excluded from care decisions about a transfer to the SNF [33]. Digby et al. who interviewed eight people living with dementia revealed they were often not consulted about their care and they felt powerless and unsettled in their new location [31]. An example cited by Emmett et al. describes the anger felt by a patient who was excluded from decision-making about her future care needs [32].

In their study of family carers and health and social care staff, Renehan et al. reported positive communication practice [36]. Six of the seven family carers felt they received adequate information, which was done by regular meetings, and contact with the Clinical Nurse Consultant prior to admission, during the stay and at point of discharge. This clinician undertook the assessments for the service. Two of the seven families reported difficulty getting hold of the clinician but families appreciated having access to a named professional. Responses from social care professionals from the discharge destination facilities were also favourable stating information was comprehensive and timely. The provision of designed documentation, which detailed a full social and medical history of the person, was greatly valued by the facility staff.

#### Quality of care

The majority of the studies noted inconsistency in quality of care and the capability of healthcare professionals to care for people living with dementia when transitioning to a different location. This often resulted in unsatisfactory experience and outcomes for all stakeholders involved.

Families expressed concerns about standards of care in hospitals, in particular with assumptions being made about the psychosocial needs of the patient, the person’s level of function, (such as maintaining activities of daily living whilst in hospital) and involvement of the family [28, 30, 35]. The people living with dementia interviewed by Digby et al. also described a lack of understanding on the part of healthcare professionals in the facility, as they reported often feeling patronised and unsettled by the lack of personal empowerment in the environment [31].

Care facility and hospital healthcare professionals reported having difficulties in caring for people living with dementia particularly those with behavioural symptoms. In one study, [34] residential care staff reported that people living with dementia often returned from hospital over-sedated, without having their original health needs addressed. They attributed this to hospital based healthcare professionals’ inability to respond to the needs of the person living with dementia. Hospital based healthcare professionals in turn felt they lacked knowledge in caring for people living with dementia and felt specialist support was lacking.

Nurses in a SNF also admitted to feeling ill-equipped to care for people living with dementia who were transferred from hospital. This they attributed to the lack of detailed information about the person’s behaviour and the supportive care which was required [33]. Some care home nurses felt that hospitals under reported behavioural symptoms. As a result the care home did not have the workforce capacity to respond appropriately to some of the needs of patients transferred from hospital. Residential care home staff also reported that when transfers happened during out of office hours, there was no registered nurse on duty [34].

Positive outcomes for people living with dementia who transferred into the specialist transition care programme were reported [36]. The quantitative results indicated a reduction in the frequency of agitated behaviours on the Cohen Mansfield Behavioural inventory compared to the scores when the person was in hospital. However, some of the data were incomplete so some caution must be exercised with interpreting this finding. There was some qualitative verification of this finding, with families reporting reduction in agitation, improved socialisation and health outcomes following transfer from hospital to the facility.

#### Carer engagement and roles of the family

All stakeholders agreed that families have an important role to play in transitions from hospitals to care facilities with all studies discussing this topic. Stakeholders noted that successful transitions occurred when family members were involved prior, during and after the transfer. Families often filled the gaps in the care system and were noted to be actively involved in providing advocacy, facilitating communication, helping with personal care, providing much needed stimulation and helping to prepare the environment [30, 32, 35]. Both Fitzgerald et al. and Digby et al. reinforced that family support and regular presence provided comfort to the person living with dementia [29, 31]. Nurses working in an SNF also noted a smoother transition when opportunities arose to work directly with the family in preparing the person for transfer to the facility [33]. Despite this recognition of the valuable role of families, they were often not consulted about discharge arrangements [28, 34] . The lack of family involvement and exclusion in decision making led family members to feel frustrated and unappreciated [32].

Moving from hospital to care home for the first time can be particularly challenging for family members. Tension and family conflict about decisions were common [30] and conflicts of interests between patients and relatives regarding funding care were noted [32]. Working with families was difficult from the healthcare professional perspective, the hospital nurses in Reneham et al’s study found supporting families a challenge due to the multiple roles that they had [36].

Family carers found the time leading up to discharge within the hospital environment, the associated decision making about moving into a care facility, the care processes and systems particularly stressful. [28–30, 32].

## Discussion

This is the first review of stakeholders’ perspectives on the transition for people living with dementia from hospital to community care settings. A striking finding is the paucity of studies (*n* = 8) eliciting stakeholders’ perspectives on this experience and on the outcomes of care. The majority of these studies were conducted in Australia and relatively recently. Despite the development of the concept of transitional care in the US, few of these studies on transitional care examine stakeholder perspectives of this transition for those living with dementia.

In this review we created a narrative synthesis of stakeholders’ perspectives on transitional care of people living with dementia moving from hospital to a care facility. We established four inter related issues of importance to all stakeholders: adequate and inclusive preparation for transition; the need for good quality and timely communication; quality of care and family carer engagement. The studies in the review have noted there are shortfalls within these four categories, from the perspective of all stakeholders, which negatively impacts on all stakeholders involved. Findings also highlight facilitators for improved experience.

Although there are reviews that have focused on hospital discharge processes for people living with dementia [37–39] this review examined additional transitional care activities with emphasis on moving to and being received by a care facility. Our findings are consistent with these reviews, in identifying some of the barriers to successful transition out of hospital for people living with dementia. Furthermore they identify specific issues relating to transferring to a care facility. It highlights a number of clinical practice issues resulting in unsatisfactory experiences, which could be improved upon.

Evidence from this review demonstrates many people living with dementia are often transferred to an alternative setting or care for either rehabilitation or to wait until a long-term care placement can be found. This has clinical implications as opinion is mixed whether multiple relocations for people with dementia causes further problems and should be minimised [40]. Whilst experiencing this alternative location in care or waiting for placement, the person with dementia and their family were often receiving care that did not meet the needs of individuals. Family members often act as advocates and provide ‘hands on’ care whilst the person is in hospital or a transition setting but conversely are not included in decision making about care. These findings are also reflected in a review of proxy decision-making by families of people living with dementia, which noted family carers were excluded from decisions made in hospital [27].

Health and social care professionals working with family members are fundamental in the care of people living with dementia. Successful transfers were reported when health and social care professionals were able to effectively engage with families. One study in this review demonstrated positive outcomes for people living with dementia when they were referred to a dementia specialist transition care program [36]. Increased family satisfaction was achieved when families had a named professional with whom they could liaise and communicate with both in the hospital and following transition into the facility. The practice of having a dedicated professional responsible for discharge planning is also supported in national UK guidance but is not specific about their role after transition [41].

Health and social care professionals also felt they did not have the skills to manage the complexities involved in caring for people with dementia. It is commonly recognised that when people with dementia are unwell or are in a place which is unfamiliar their response may be one of heightened anxiety and agitation [42], yet examples were given of health care professionals not being able to respond to this with non-pharmacological approaches and resorting to medication. This is not unusual, studies have reported that care of people living with dementia in hospital is often task orientated, delivered with little social engagement, sometimes with a lack of compassion and inappropriate responses to agitated behaviours such as the use of security services [43]. Given that this review has identified many people living with dementia often receive short-term care due to delays in arranging care to their final destination, it is important that staff in these areas have the skills to provide competent care.

System pressures resulting in sudden transfers were often a barrier to providing sufficient information between settings. Assumptions were made by hospital based healthcare professionals that facilities could respond quickly with little appreciation of the time required to prepare the right environment and arrange supplies of essential medication. Similar findings were noted in a Swedish study [44] that examined hospital and care home nurses’ views of older people transfers between settings. It found that transfers back to nursing homes were commonly done with limited planning, late in the day with little communication between nurses in either setting.

The emphasis on rapid turnover of patients is also not conducive to the patient with dementia. Kitwood’s theory of person centred dementia care [45] and the need to prevent behaviours such as outpacing and de-personalised care regimes suggests that such hurried transfers with little preparation of the person can significantly undermine a person’s well-being. System pressures also place staff under considerable stress. Findings of this review echo those in an English study by Connolly et al. [46], who reported practitioners were conflicted by competing internal and external pressures, which undermined their professionalism, caused frustration, and resulted in de-personalisation of care.

These findings have noted that how discharge activities are implemented can have unintended consequences for the care the person receives at the care facility. There is a sense in the literature that systems and processes between settings can be adversarial. An appreciation of perspectives of both ends of the pathway, and a focus on working together may help to facilitate better transitional care experiences.

### Limitations of the included studies

There are some limitations of this review which need to be considered when interpreting the findings. The studies reviewed used limited methods of data collection, mostly focus groups and interviews, although these methods yield rich data, the researchers are relying solely on the interpretation of the participants. Only two studies used supplementary data collection methods. Observational methods were particularly lacking, and one of the reasons may be the practicality of being able to observe transitional care in practice. Most studies used purposive sampling, relying on volunteers to come forward to participate in research, their views may not represent those who did not volunteer and may be skewed due to having negative experiences of the transition. A further limitation is the variation in the care facilities that were studied. There was a range of providers from government funded, to privately owned profit and non-profit facilities. Operational and clinical procedures, staff ratios and training are likely to be varied. Additionally all the studies were from a limited number of western countries with different health care systems and findings may not be transferable to other healthcare contexts.

### Limitations of this review

Although a comprehensive search was undertaken from six databases and hand-searching only eight studies were found in this review. More studies from grey literature and non-peer reviewed journals may exist, but not necessarily of high quality. This does limit the conclusions that can be drawn.

## Conclusions

The findings point to this key transition for people living with dementia being an under-researched area fraught with clinical care concerns. Despite the policy focus on person-centred care few studies seek the perspective of people living with dementia and their families. Yet we found that diverse stakeholders share common concerns about care during this transition including the need for: adequate preparation for transfer; effective communication between settings and stakeholders, quality care whilst their long term care needs are arranged and family engagement throughout. We identified areas of best practice which can help to guide care. There is a compelling need for further research in order to improve the experiences of the transition from hospital to care facilities for people living with dementia, their families and health care professionals.

## Data Availability

N/A

## Abbreviations

9pt?>ALC: Alternative level of care
HCP: Health and social care practitioners
PLWD: People living with dementia
SNF: Skilled nursing facility
TC-CAMP: Transition care cognitive assessment and management pilot
UK: United Kingdom
US: United States of America
